# Why Does the Type of Halogen Atom Matter for the Radiosensitizing Properties of 5-Halogen Substituted 4-Thio-2′-Deoxyuridines?

**DOI:** 10.3390/molecules24152819

**Published:** 2019-08-02

**Authors:** Paulina Spisz, Magdalena Zdrowowicz, Samanta Makurat, Witold Kozak, Konrad Skotnicki, Krzysztof Bobrowski, Janusz Rak

**Affiliations:** 1 Laboratory of Biological Sensitizers, Faculty of Chemistry, University of Gdańsk, Wita Stwosza 63, 80-308 Gdańsk, Poland; 2 Centre of Radiation Research and Technology, Institute of Nuclear Chemistry and Technology, Dorodna 16, 03-195 Warsaw, Poland

**Keywords:** radiosensitizers, stationary radiolysis, pulse radiolysis, modified nucleosides, cellular response

## Abstract

Radiosensitizing properties of substituted uridines are of great importance for radiotherapy. Very recently, we confirmed 5-iodo-4-thio-2′-deoxyuridine (ISdU) as an efficient agent, increasing the extent of tumor cell killing with ionizing radiation. To our surprise, a similar derivative of 4-thio-2’-deoxyuridine, 5-bromo-4-thio-2′-deoxyuridine (BrSdU), does not show radiosensitizing properties at all. In order to explain this remarkable difference, we carried out a radiolytic (stationary and pulse) and quantum chemical studies, which allowed the pathways to all radioproducts to be rationalized. In contrast to ISdU solutions, where radiolysis leads to 4-thio-2’-deoxyuridine and its dimer, no dissociative electron attachment (DEA) products were observed for BrSdU. This observation seems to explain the lack of radiosensitizing properties of BrSdU since the efficient formation of the uridine-5-yl radical, induced by electron attachment to the modified nucleoside, is suggested to be an indispensable attribute of radiosensitizing uridines. A larger activation barrier for DEA in BrSdU, as compared to ISdU, is probably responsible for the closure of DEA channel in the former system. Indeed, besides DEA, the XSdU anions may undergo competitive protonation, which makes the release of X^−^ kinetically forbidden.

## 1. Introduction

Trojan horse radiotherapy employs a nucleoside radiosensitizer, a “Trojan horse”, that is activated only due to DNA exposure to ionizing radiation [1]. Such radiosensitizers are usually electrophilic nucleosides, incorporated into DNA during replication and repair, and undergoing efficient dissociative electron attachment (DEA) that leaves behind a nucleoside radical, which in secondary reactions is able to produce damage to the biopolymer (frequently a strand break) [1]. Although the purine derivatives of nucleosides were proposed as potential radiosensitizers [2,3,4,5], most of the reported examples comprise uridines substituted at the C5 position. This is because thymidine kinase accepts a broad set of modified uridines [6], which after phosphorylation may be incorporated into DNA [7]. The modified DNA sensitivity to hydrated electron attachment is especially important for radiotherapy, since cells of solid tumors (80% of cases [8]) are hypoxic, which make them resistant to hydroxyl radicals, a major damaging agent of native DNA produced during radiotherapy [9]. In the normoxic cells, damage produced by the ^●^OH radicals becomes “fixed” due to reaction with oxygen, while under hypoxia, naturally occurring radioprotectors like cysteine or glutathione can restore DNA through hydrogen donation [10]. Moreover, hydrated electrons, the second most abundant product of water radiolysis, are not harmful to the natural DNA [11,12]. The situation becomes quite different when DNA is labeled with nucleosides undergoing efficient DEA. Indeed, Sanche et al. [13] demonstrated efficient formation of single strand breaks in DNA oligonucleotides labeled with BrdU, when an aqueous solutions of these biopolymers were irradiated with X-rays in the presence of a hydroxyl radical scavenger. Similarly, a radiolysis of a solution containing TXT oligonucleotides (where X stands for 5-bromo-2′-deoxyuridine (BrdU), 5-iodo-2′-deoxyuridine (IdU), 5-bromo-2′-deoxycitidine (BrdC), 5-iodo-2′-deoxycitidine (IdC), 8-bromo-2′-deoxyadenosine (BrdA), or 8-bromo-2′-deoxyguanosine (BrdG)) and *t*-butyl alcohol (*t*-BuOH) as ^●^OH scavenger led to strand breaks besides other types of DNA damage [11,12]. 

BrdU and IdU are well-known radiosensitizers, which are phosphorylated in cytoplasm forming the respective 5′-triphosphates and then incorporated into the cellular DNA by human DNA polymerases [7]. Their promising radiosensitizing properties were investigated in numerous in vitro [14,15,16] and in vivo [17] studies and even in clinical trials [18]. In one of the most extensive clinical studies on brain tumor patients, no positive effects were observed in patients exposed to the specific doses of BrdU besides radiotherapy [18]. To this end, it is worth emphasizing that a swift and efficient metabolism (most radiosensitizers, as other chemotherapeutics, are applied systemically) of a sensitizer, may lead to its lower cellular concentration in vivo than in vitro. This, at least partially, explains a high radiosensitizing activity of BrdU in vitro, and practically, the lack of such activity in the clinical studies. 

This situation calls for new radiosensitizers of superior pharmacokinetic and/or better radiosensitizing properties. Recently, we proposed several new C5-pyrimidine derivatives that have not been studied in animal models or in clinic to date [19,20,21,22,23]. In terms of electron-induced degradation yields, they are all more prone to dissociative electron attachment (DEA) than BrdU. These compounds comprise 5-thiocyanato-2′-deoxyuridine (SCNdU), 5-selenocyanato-2′-deoxyuridine (SeCNdU), 5-selenocyanatouracil (SeCNU), and 5-trifluoromethanesulfonyl-2′-deoxyuridine (OTfdU). Other promising candidates of this type of radiosensitizers seem to be derivatives of 4-thio-2′-deoxyuridine. The latter compound, similarly to BrdU and IdU, is incorporated into genomic DNA by the cellular enzymatic machinery [24]. It is worthwhile to note that BrdU works both as a DNA radio- and photosensitizer [1]. On the other hand, the photosensitizing properties of BrSdU and ISdU were proven in the past [25,26]. By the same token, one may conclude that 5-haloderivatives of 4-thio-2′-deoxyuridine could also work as radiosensitizers. Indeed, we recently demonstrated the radiosensitizing properties of ISdU [27]. Under the same conditions, the yield of damage produced by 140 Gy of X-ray was 1.5-fold larger than that assayed in the irradiated BrdU aqueous solutions. Simultaneously, in vitro studies demonstrated a significant increase of the mortality in cells treated with ISdU after irradiation.

In the current paper, studies on BrSdU–similar to those shown in [27] on ISdU–are described. To our surprise, BrSdU does not possess increased radiosensitizing properties. It is decomposed during radiolysis by X-ray, but the stable products resulted only from the reactions between the compound studied and H_2_O_2_ or radicals forming in the reaction between *t*-BuOH and the ^●^OH radicals. We did not observe the characteristic pattern of DEA, i.e., the formation of 4-thio-2′-deoxyuridine, in this case. In accordance with this finding, the clonogenic assay does not differentiate the cells that were grown with and without BrSdU. We explain this striking difference between ISdU and BrSdU with the height of activation barrier for DEA, which is almost twice as much as in BrSdU.

## 2. Results and Discussion

A radiosensitizing nucleoside working under hypoxia must be sensitive to hydrated electrons, which are the second most abundant product of water radiolysis. In order to assess the radiosensitizing potential of a nucleoside, one must expose its aqueous, deoxygenated solution to ionizing radiation. If hydroxyl radicals are scavenged during irradiation, only the reaction between hydrated electrons and potential radiosensitizer may lead to serious damage associated with the formation of radical products. If the radiolysis proceeds in the cells containing DNA labeled with radiosensitizer, those radicals will produce DNA damage, hopefully single/double strand breaks, leading to apoptosis as an ultimate cellular response. This is why the radiolysis of a nucleoside in aqueous solution and the qualitative and quantitative analysis of radiolytic products is an indispensable step for assessing the radiosensitizing potential of the derivative under investigations. 

### 2.1. Stationary Radiolysis

**Figure 1:**
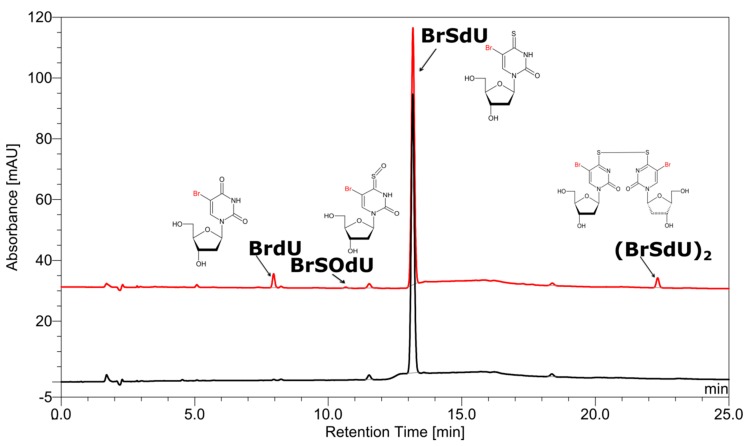
High-performance liquid chromatography (HPLC) traces for a solution of BrSdU before (black) and after irradiation (red). The chemical structures of products, as indicated by the liquid chromatography–mass spectrometry (LC–MS/MS) analysis, are depicted at particular peaks.

The high-performance liquid chromatography (HPLC) traces of radiolytes, originating from X-ray irradiation of buffered aqueous solution, containing 10^−4^ M of BrSdU in the presence of a hydroxyl radical scavenger (*t*-BuOH, 0.03 M) with the dose of 140 Gy, are depicted in Figure 1. 

**Figure 2:**
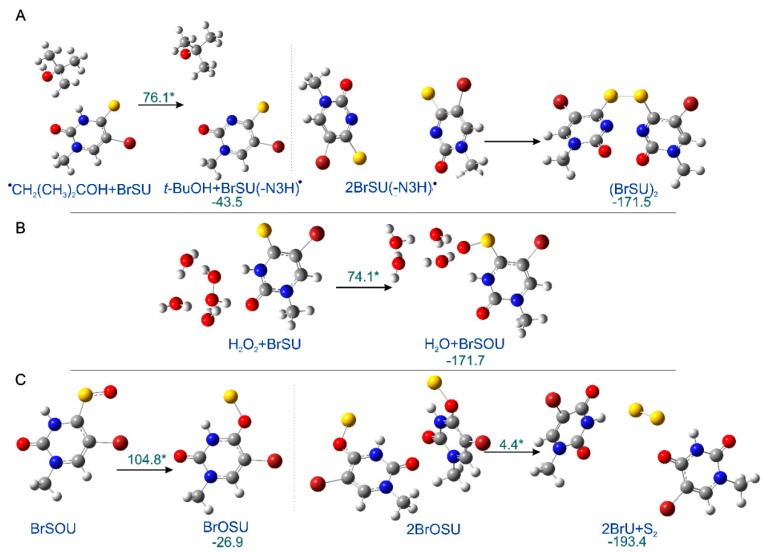
The radiolysis products. (**A**) (BrSU)_2_, (**B**) BrSOU, and (**C**) BrU formation, as suggested by calculations. The optimized reactants in ball and stick representation are shown along with their kinetic barriers (marked with asterisks) and thermodynamic stimulus (kJ/mol). All the reactions shown correspondence to the most favorable pathways, as obtained by the IRC procedure. The transition states structures can be found in Supplementary Materials (Figure S5).

As indicated by the comparison of two chromatograms (Figure 1), three main products are formed due to radiolysis. The liquid chromatography–mass spectrometry (LC–MS) analysis enabled the identification of all these species. Figure 1 also depicts the chemical structures of the identified decomposition products, while the MS/MS spectra (shown in Figures S1–S4 in Supplementary Materials) confirm the assignment of particular structures. These radiolysis products are a dimer and two oxidation products, whose probable mechanism of formation was modelled for 5-bromo-1-methyl-4-thiouracil (see the Computational section) at the B3LYP(PCM)/DGDZVP++ level and is shown in Figure 2. The (BrSU)_2_ dimer is suggested to be the product of two BrSU^•^ radicals recombination (Figure 2A), which are created in the reaction of BrSU with ^•^CH_2_(CH_3_)_2_COH (the ^•^CH_2_(CH_3_)_2_COH radicals are formed in the reaction between *t*-BuOH, present in the solution as a hydroxyl radical scavenger, and the ^•^OH radicals–a primary product of water radiolysis). The reaction is associated with a kinetic barrier of 76.1 kJ/mol and is favorable thermodynamically (Figure 2A). The second reaction, leading to BrSOU (Figure 2B) due to oxidation of BrSU by H_2_O_2_ [28,29,30] (H_2_O_2_ is produced during water radiolysis [31]), was modelled with three explicitly added water molecules, which is the approach that had been suggested in the literature [32]. It is worthwhile to note that under the experimental conditions, BrSOU (BrSOdU) appears to be the least abundant product, which probably results from the fact that it is also the substrate for the most abundant one, i.e., for BrU (Figure 2C). The latter is formed via a cyclic oxathiirane followed by the sulfur extrusion reaction [33,34]. In our calculations, we were unable to obtain the stable oxathiirane intermediate. During the optimization, the ring opened to give the BrOSU structure shown in Figure 2C. Also the sulfur extrusion does not show any intermediates. After the TS structure is achieved (4.4 kJ/mol barrier), one of the sulfur atoms attaches to the other one and the S-O bond breaks leading to BrU and S_2_ (Figure 2). 

To our surprise, one of the expected products, 4-thio-2′-deoxyuridine, that should form due to DEA to BrSdU, was not detected in the BrSdU radiolytes (see Figure 1). DEA, occurring in many similar systems including BrdU, IdU, and ISdU, is thought to be the main reason of DNA damage in the irradiated cells, i.e., it is responsible for the radiosensitizing potential of modified nucleosides [12,35,36,37]. However, neither 4-thio-2′-deoxyuridine nor dimer with the substrate (both were observed in radiolytes of ISdU) were observed among the radiolysis products. 

**Figure 3:**
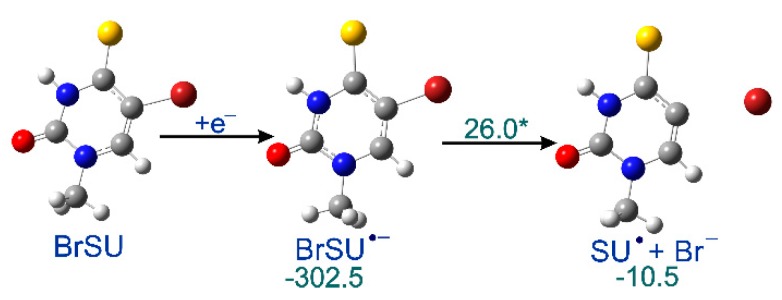
Calculated dissociative electron attachment (DEA) profile for BrSU. After the initial electron attachment to BrSU, the anion radical BrSU^•−^ is formed, and subsequently dissociates via a transition state giving SU^•^ and Br^−^. The thermodynamic and kinetic characteristics [kJ/mol], shown in green, were calculated as the difference between the given state and the previous stable one, the transition state barrier marked with asterisk.

In order to explain why the radiolysis of ISdU leads to SdU, while this reaction channel is actually closed for BrSdU under the same experimental conditions, we calculated the respective DEA profile (Figure 3). We found that the kinetic barrier for the C5-Br bond had breakage as much as 26.0 kJ/mol–more than two times higher than that for C5-I in ISU (12.6 kJ/mol [27]) dissociation calculated at the same level of theory. Similarly, the thermodynamic stimulus for the release of the bromide anion from BrSU^•−^ amounts to only −10.5 kJ/mol as compared to −24.3 kJ/mol for ISU^•−^ [27]. These differences, especially a significant difference in the height of activation barriers in the two compared systems, explains formation of SdU only in the latter derivative. The estimated lifetime of BrSdU^•−^ at the ambient temperature is ca. 200-fold longer than that of ISU^•^, which results from the above-mentioned activation barriers and transition state theory [38]. It is probably sufficiently long to allow the anion to be protonated, which prevents completion of the DEA process [39], and in consequence, SdU is not formed. 

### 2.2. Pulse Radiolysis

**Figure 4:**
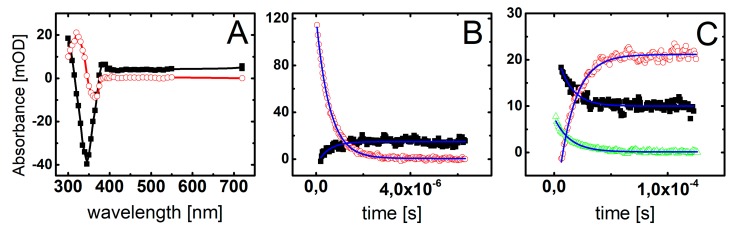
(**A**) Transient absorption spectra recorded in deoxygenated and buffered with phosphate (10 mM, pH = 7.0) ISdU solution (5 · 10^−5^ M), in the presence of 0.5 M *t*-BuOH, after 2 μs (■) and 120 μs (◯) after the electron pulse. (**B**) Short-time profiles representing the growth at λ = 305 nm (■) and the decay at λ = 720 nm (◯) of transient absorptions and their least-square fits to the first order formation and decay, respectively. (**C**) Long-time profiles representing the growth at λ = 320 nm (◯) and decays at λ = 300 nm (△) and λ = 385 nm (■) of transient absorptions and their least-square fits to the first order formation and decays, respectively.

**Figure 5:**
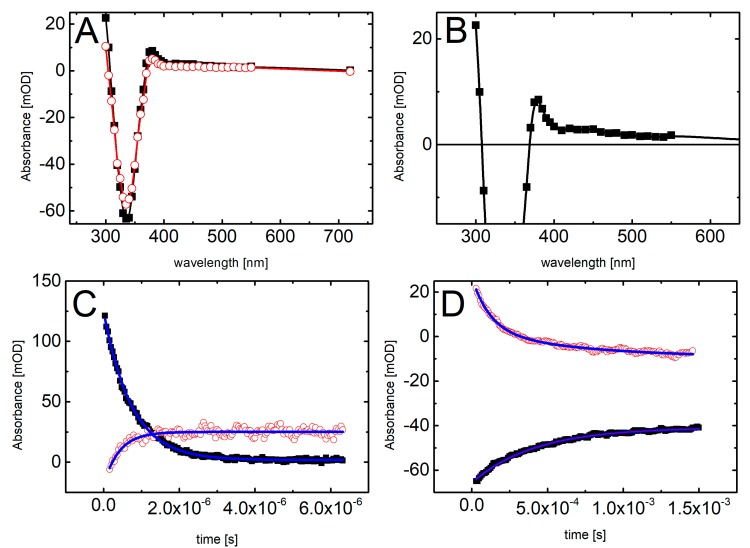
Transient absorption spectra recorded in deoxygenated and buffered with phosphate (10 mM, pH = 7.0) BrSdU solution (5 · 10^−5^ M), in the presence of 0.5 M *t*-BuOH (**A**) after 12 μs (■) and 120 μs (◯) and (**B**) (■) 12 μs after the electron pulse. (**C**) Short-time profiles representing the growth at λ = 300 nm (◯) and the decay at λ = 720 nm (■) of transient absorptions and their least-square fits to the first order formation and decay, respectively. (**D**) Long-time profiles representing the growth at λ = 335 nm (■) and decays at λ = 300 nm (◯) of transient absorptions and their least-square fits to the second order formation and decays, respectively.

The hypothesis, explaining different behavior of BrSdU and ISdU based on the computational results and discussed in the previous section, is confirmed by the results of our pulse radiolysis experiments. Figure 4 and Figure 5 depict the transient spectra, as well as the respective decays and growths in microsecond-time domain for ISdU and BrSdU, respectively. 

The resulting transient spectrum, obtained 2 μs after the electron pulse, exhibits a rising absorption toward 300 nm with no defined maximum and negative absorption in the wavelength range 320–380 nm (Figure 4A). This time delay for spectra recording was chosen on purpose in order to get rid of participation of hydrated electrons in the spectrum. Nonetheless, the registration of “pure” spectrum of this transient product was not possible due to the bleaching related to the consumption of ISdU. The decay at λ = 720 nm represents the decay of hydrated electrons in the presence of 5 · 10^−5^ M ISdU with the pseudo-first order rate constant *k*_720_ = 1.8 · 10^6^ s^−1^. In turn, the growth at λ = 300 nm represents the formation of a transient product with the pseudo-first order rate constant *k*_300_ = 2.0 · 10^6^ s^−1^ (Figure 4B). Since these rate constants are very similar, this species could be a direct product of the hydrated electron attachment to ISdU if the lifetime of ISdU^●−^ was long enough and included the microsecond-time domain. However, as indicated by the B3LYP/DGDZVP++ barrier height, its lifetime should be very short and is rather in the nanosecond-time domain (see the previous section). Therefore, we probably observed the product of DEA to ISdU, i.e., the SdU^•^ radical formed via Reaction (1).

(1) $$e_{aq}^{-}\;+\;\mathrm{ISdU}\;\to \;ISdU^{•-}\to SdU^{•}+\;I^{-}$$

Indeed, the calculated UV spectrum for SdU^•^ is characterized by λ_max_ located at 300 nm. With the time elapsed, the absorption spectrum underwent further changes and 120 μs after the electron pulse is characterized by a transient absorption band with λ_max_ = 320 nm, which can be assigned to a new product (Figure 4A). The growth at λ = 320 nm is mono-exponential and occurs with the pseudo-first order rate constant *k*_320_ = 7.0 · 10^4^ s^−1^. Interestingly, the decays at λ = 300 nm and 385 nm are also mono-exponential, with the respective pseudo-first order rate constants *k*_300_ = 8.2 · 10^4^ s^−1^ and *k*_385_ = 7.1 · 10^4^ s^−1^, which are reasonably close to *k*_320_ (Figure 4C). Since these decays seem to represent the decay of the SdU^•^ radical, the growth observed at λ = 320 nm can be tentatively assigned to the formation of SdU via reaction of SdU^•^ with hydrogen atom donor, which is *t*-BuOH present in the system in a large excess (Reaction 2).

(2) $${\mathrm{SdU}}^{•}+\;t\text{-}\mathrm{BuOH}\;\to \;\mathrm{SdU}\;+\;{\;}^{•}\mathrm{CH}2(\mathrm{CH}3)2\mathrm{COH}$$

The calculated (and measured) UV spectrum of 4-thiouracil strongly supports this assignment. Moreover, the absorption at λ = 320 nm is stable in the time window of our experiment (up to 1.5 ms) with no signs of disappearance, which might suggest, however not directly, a high stability of the product.

The main difference between spectral features observed during pulse radiolysis of ISdU and BrSdU aqueous solutions is the lack of the transient absorption band with λ_max_ = 320 nm for the latter system (compare Figure 5A with Figure 4A). This finding clearly shows that the formation of SdU does not occur in BrSdU via analogous reaction 2, which requires the presence of SdU^•^ radical. This fact remains in a good accordance with the results of stationary **γ**-radiolysis, where we did not observe the product of the bromide anion (Br^−^) release from BrSdU^•−^. In case of ISdU, we also observed an increase in the integrated XIC signal of the iodide anions (a background signal of iodide anions is observed due to synthesis-related contamination, unavoidable degradation of the sample in an aqueous solution, as well as fragmentation of the studied nucleosides in the MS source [40,41]) due to stationary radiolysis. Indeed, the ratio of the signals after and before irradiation is equal to 2.041 ± 0.016. When it comes to the BrSdU sample, the integrated XIC signal of Br^−^ anions is the same before and after irradiation, 0.987 ± 0.0791, within the error bar. Using the calibration curve (not shown), we found out that the measured increase in the concentration of I^−^ due to irradiation means that ca. 27% of ISdU decay occurs in the DEA pathway. Thus, the observed changes in the concentration of halogen anions support that DEA process is operative only in ISdU solutions. This fact can be rationalized by the slower dissociation of BrSdU^•−^ (Reaction 3) in comparison to dissociation of ISdU^•−^, which allows BrSdU^•−^ to be involved in another competitive process, for instance, its protonation by water or phosphate anions (Reaction 4):

(3) $$BrSdU^{•-}\to SdU^{•}+\;Br^{-}$$

(4) $$BrSdU^{•-}\overset{H_{2}O,\;\;H_{2}PO_{4}^{-}}{\to }BrSdUH^{•}$$

The resulting absorption spectrum, obtained 12 μs after the electron pulse, exhibits a rising absorption toward 300 nm with no defined maximum and negative absorption in the wavelength range 320–380 nm (Figure 5A). This time delay for spectra recording was again chosen on purpose in order to get rid of participation of the most hydrated electrons in the spectrum. Since the absorption spectrum recorded 120 μs after the electron pulse does not exhibit the absorption band with λ_max_ = 320 nm (in contrast to ISdU), the absorption spectrum recorded 2 μs after the electron pulse cannot consequently be assigned to SdU^•^ radical. Moreover, it is worthwhile to note that the pseudo-first rate constant of the decay of hydrated electrons in the presence of 5 · 10^−5^ M BrSdU (k_720_ = 1.4 · 10^6^ s^−1^) is nearly two-fold lower than the pseudo-first order rate constant of the formation of the transient measured at λ = 300 nm (k_300_ = 2.4 · 10^6^ s^−1^) (Figure 5C). This observation suggests that this transient product cannot be formed in an analogous Reaction (2), as observed for ISdU, but might result from the protonation of BrSdU^•−^ (Reaction 4) (*vide supra*). Therefore, the absorption spectrum recorded after 12 μs can be tentatively assigned to BrSdUH^•^. The UV-VIS spectrum of BrSdUH^●^ (calculated by us) possesses the absorption bands with λ_max_ = 280, 350, and 480 nm. Therefore, for better visualization of the experimental spectrum assigned by us to BrSdUH^•^ radical, the spectrum recorded 12 μs after the pulse was zoomed to see expected spectral features (Figure 5B). The transient absorption depicted in Figure 5B shows the maxima below 300 nm and at 450 nm. The maximum at 350 nm is concealed by the “negative” absorption of BrSdU, but a tail at 380 nm is quite clear.

Interestingly, the decay at λ = 300 nm and the formation at λ = 335 nm within 1.5 ms time domain can be fitted by the second-order kinetics and occur with the respective second-order rate constants 2k_300_ = 1.2 · 10^9^ M^−1^s^−1^ and 2k_335_ = 1.0 · 10^9^ M^−1^s^−1^, which are very similar (Figure 5D). One of the processes, which can be described by the second-order kinetics is disproportionation reaction involving BrSdUH^•^ radicals, should lead to the partial recovery of the substrate (BrdSU) (Reaction 5).

(5) $$BrSdUH^{•}+\;\;\;BrSdUH^{•}\;\;\to \;\;\;BrSdU\;+BrSdUH_{2}$$

Thus, the decay observed at λ = 300 nm represents the decay of BrSdUH^•^ radicals and the growth observed at λ = 335 nm represents recovery of BrSdU substrate. This mechanism can, at least in part, explain lower consumption of BrSdU compared to ISdU using the same dose delivered by X-rays.

### 2.3. Biological Assessments

#### 2.3.1. Incorporation of BrSdU and ISdU into Genomic DNA

According to the concept of the Trojan horse therapy, a radiosensitizer should easily incorporate into DNA. For this reason the incorporation of BrSdU and ISdU into genomic DNA was assessed. The MCF-7 cells treated with BrSdU and ISdU at the concentration of 10^−4^ M were incubated for 48 h. Purified DNA was enzymatically digested and analyzed by HPLC (Figure S6) and LC-MS method. The results of LC-MS analysis shows that both derivatives incorporate to DNA (see extracted-ion chromatograms, MS and MS/MS spectra for BrSdU/ISdU in Figures S7–S10 in Supplementary Materials), but the efficiency of this process is very low, which has been already observed by others [24]. To our surprise, BrdU and IdU beside BrSdU and ISdU were also observed in the digested material, which suggests an enzymatic degradation of BrSdU/ISdU to BrdU/IdU in the cell with the rate similar to that of BrSdU/ISdU incorporation into DNA. 

#### 2.3.2. Clonogenic Assay 

**Figure 6:**
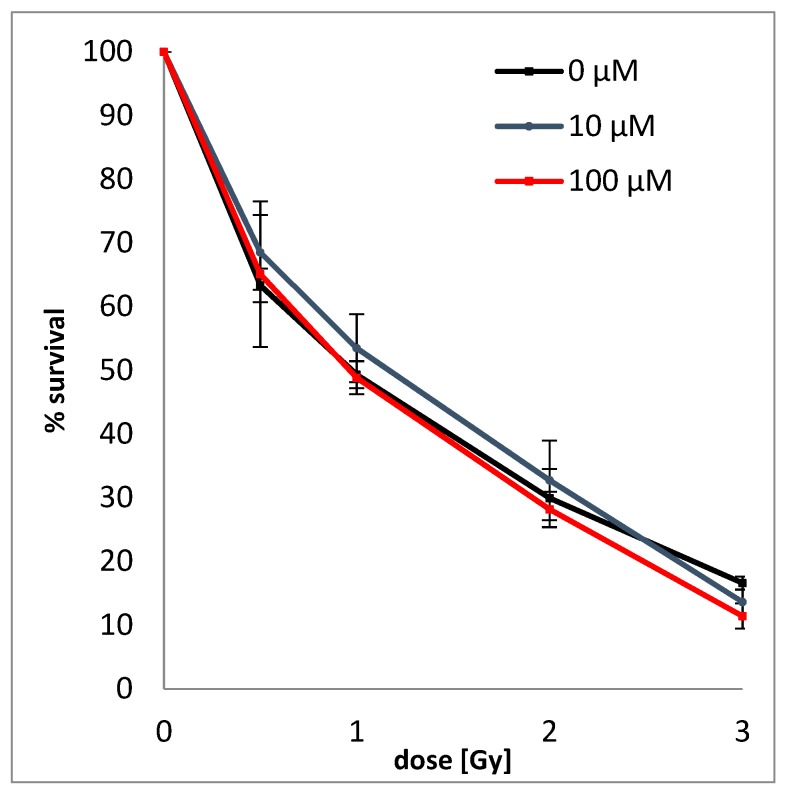
Dose response curves of MCF-7 cells treated with (10 µM or 100 µM solutions of BrSdU) or without BrSdU. The average plating efficiencies for the controls with and without pretreatment are equal to 28.16% (0 µM), 25.63% (10 µM), and 24.78% (100 µM). Experiments were performed at least in two independent experiments in duplicate and the results are expressed as mean ± standard deviation.

In order to determine the simultaneous effect of BrSdU and ionizing radiation on the survival and proliferation of cancer cells, the clonogenic assay (based on the ability of a single cell to grow into a colony) [42] was performed. The test was carried out on human breast cancer cells (MCF-7 line) treated with BrSdU at the concentration of 0, 10, and 100 µM and/or ionizing radiation (IR) in four doses of 0.5, 1, 2, and 3 Gy. Figure 6 shows that the studied compound does not affect the survival of cancer cells. We only observed reduction of survival fraction caused by IR. For example, in case of dose of 2 Gy, survival was 29.9 ± 4.6%, 32.7 ± 6.3%, and 28.2 ± 2.8% for 0, 10, and 100 µM BrSdU pretreatment, respectively. This colony formation assay demonstrates that the BrSdU does not sensitize the MCF-7 cells to X-ray. In our previous studies, we demonstrated a significant radiosensitizing effect of ISdU [27]. The addition of the latter derivative to cell culture resulted in a significant decrease (about 20%) of their survival after irradiation, even with doses as low as 0.5 Gy. 

#### 2.3.3. Cytotoxicity Assay

**Figure 7:**
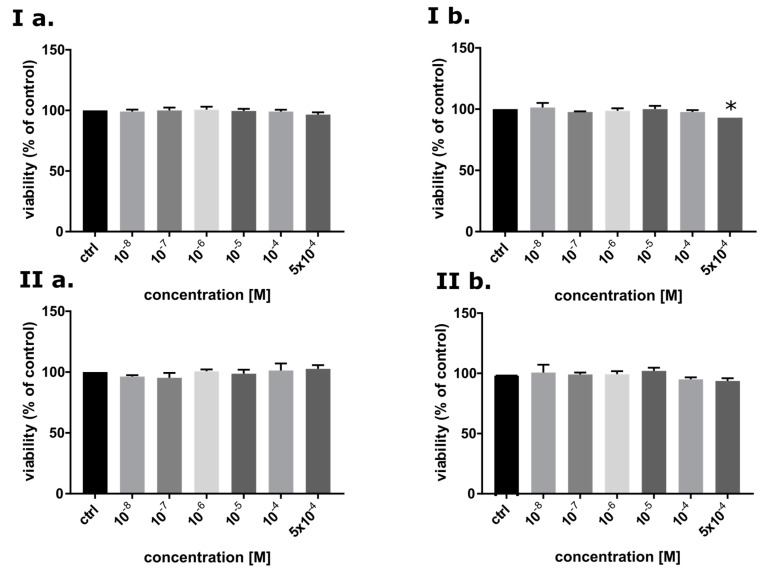
The viability of MCF-7 (**I**) and HDFa (**II**) cells after 24 (**a**) and 48 h treatment (**b**) with BrSdU in a range of concentrations from 0 to 5 · 10^−4^ M. Results are shown as mean ± SD of three independent experiments performed in triplicate. *statistically significant difference is present between treated culture compared with control (untreated culture).

One of the properties that good radiosensitizer should possess is low cytotoxicity without IR treatment. To identify cytotoxicity of BrSdU toward MCF-7 (cancer cells) and HDFa (normal cells) line, MTT assay [43] was carried out (Figure 7). BrSdU was tested at six concentrations (0, 10^−8^, 10^−7^, 10^−6^, 10^−5^, 10^−4^, 5 · 10^−4^M) and two time variants (24 and 48 h of incubation). Figure 7Ib shows statistically significant reduction of viability up to 93% (for 48 h incubation) for MCF-7 line only in case of the highest tested concentration equal to 5 · 10^−4^ M. For lower concentrations, the decrease in vitality was not statistically significant. We also did not observe a meaningful difference between the studied cell lines. These results show that the cytotoxicity of BrSdU is very low both for normal human dermal fibroblasts and human breast cancer cells.

#### 2.3.4. Analysis of Histone H2A.X Phosphorylation and Cell Death

**Figure 8:**
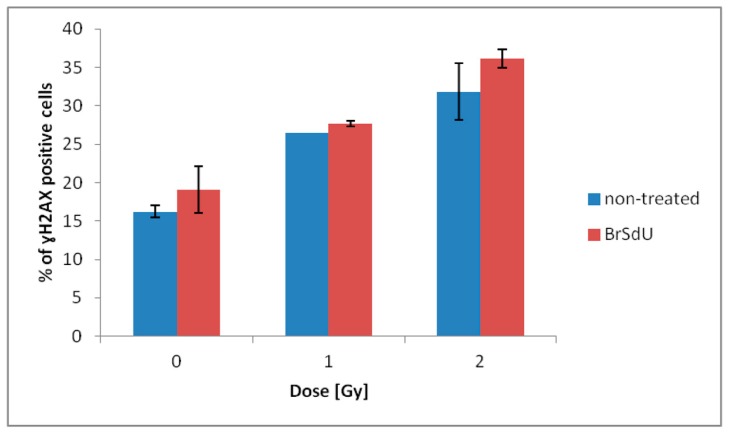
Flow cytometric analysis of H2A.X phosphorylation. γH2A.X was measured 1 h after irradiation. Results are shown as the mean ± standard deviation of at least two independent flow cytometry experiments.

One of the most common types of DNA damage related to radiosensitization is double-strand breaks formation. Phosphorylation of histone γH2A.X is the marker of such a damage [44]. The assay was performed for human breast cancer cells treated with BrSdU at concentration of 10^−4^ M and/or irradiated with a dose of 0, 1, or 2 Gy. Analysis of H2A.X phosphorylation was carried out 1 h after irradiation (this time was optimized in previous experiments). The cells were fixed and analyzed by flow cytometry. Our studies show that treatment with BrSdU results in a tiny increase in the population of γH2A.X positive cells after irradiation with the doses of 1 and 2 Gy (Figure 8 and Figure S11). After BrSdU pretreatment and irradiation with the dose equal to 1 Gy, the level of γH2A.X was changed from 26.48% (nontreated cells) to 27.64 ± 0.34%). The exposure of treated cells to the dose of 2 Gy results in a slight enhancement of the γH2A.X fraction from 31.84 ± 3.74% to 36.15 ± 1.25%.

Additionally, we performed the multidimensional test to quantify the number of viable, early apoptotic, late apoptotic, and dead cells. Our results (Figures S12 and S13 in Supplementary Materials) confirm that pretreatment with BrSdU does not affect the sensitivity of MCF-7 cells to ionizing radiation. A significant influence of the studies analog on population of viable, early apoptotic, late apoptotic, and dead cells was not observed. In the case of ISdU, the situation was completely different. The histone H2A.X phosphorylation test showed that ISdU sensitized breast cancer cells to ionizing radiation, at least in part, by formation of DSBs, while the cell death assay confirmed that pretreatment of culture with ISdU led to the IR-induced reduction of cell viability and increase in the population of early apoptotic cells [27].

## 3. Materials and Methods 

### 3.1. Chemicals

5-bromo-2′-deoxyuridine, acetic anhydride, P_2_S_5_, 1,4-dioxane, and sodium hydride were commercially available from Sigma–Aldrich (Saint Louis, MO, USA). Nuclear magnetic resonance (NMR) spectrum was recorded on a Bruker AVANCE III (Bruker, Billerica, MA, USA), 500 MHz spectrometer. Chemical shifts are reported in ppm relative to the residual solvent peak (DMSO-*d*_6_ 2.49 ppm for ^1^H and 39.5 ppm for ^13^C). Column chromatography was performed using silica gel NORMASIL 60 (40–63 mesh, VWR Chemicals, Gdańsk, Poland). Preparative thin-layer chromatography was performed with silica gel plates, 60G, F254 (Sigma–Aldrich).

### 3.2. Synthesis of 3′,5′-di-O-acetyl-5-bromo-2′-deoxyuridine

A solution of 5-bromo-2′-deoxyuridine (500 mg, 1.63 mmol) in pyridine (6 mL) was stirred at room temperature with acetic anhydride (339 µL, 3.59 mmol) for 24 h. The sirupus residue was co-evaporated with three portions of aqueous ethanol (5 mL) and *n*-heptane to remove the pyridine residue. The raw 3′,5′-di-*O*-acetyl derivative (580 mg) was obtained in a 91% yield.

### 3.3. Synthesis of 3′,5′-di-O-acetyl-5-bromo-4-thio-2′-deoxyuridine

3′,5′-di-*O*-acetyl-5-bromo-2′-deoxyuridine (580 mg, 1.48 mmol) was dissolved in 1,4-dioxane (20 mL) and P_2_S_5_ (990 mg, 4.45 mmol) was added. The mixture was refluxed until thin-layer chromatography (TLC) analysis (CHCl_3_:CH_3_OH, 30:1) showed complete disappearance of the substrate (2–3 h). Solvent was removed under reduced pressure and the residue was treated several times with CHCl_3_. The combined chloroform extracts were evaporated, and the residue was separated on silica gel column, which was eluted with CHCl_3_:CH_3_OH, 20:1. After evaporation, the desired product was obtained as a yellow solid (567 mg, 94%).

### 3.4. Synthesis of 5-bromo-4-thio-2′-deoxyuridine

3′,5′-di-*O*-acetyl-5-bromo-4-thio-2′-deoxyuridine (567 mg, 1.39 mmol) was dissolved in methanol (10 mL) and stirred at 0 °C. A methanolic sodium methoxide solution (111 mg, 2.78 mmol), freshly prepared from NaH and anhydrous methanol, was added in portions. The mixture was stirred at room temperature until TLC analysis showed complete disappearance of the substrate (10 min). The mixture was purified on silica gel column, which was eluted with CHCl_3_:CH_3_OH, 20:1. The final product, 5-bromo-4-thio-2′-deoxyuridine, was obtained as a yellow solid (185 mg, 41%).

^1^H NMR (Bruker AVANCE III, 500 MHz, DMSO), δ: 13.09 (s, 1H), 8.53 (s, 1H), 6.03 (t, 1H), 4.25 (q, 1H), 3.82 (q, 1H), 3.55–3.69 (m, 2H), 2.14–2.26 (m, 2H); ^13^C NMR (125 MHz, DMSO), δ: 186.8, 147.6, 137.7, 107.1, 88.2, 86.1, 69.9, 60.8, 40.8. HRMS (TripleTOF 5600+, SCIEX), *m*/*z*: [M–H]^−^ calculated for C_9_H_11_BrN_2_O_4_S 323.1636, found 322.9918; UV spectrum (water), λmax: 345 nm.

### 3.5. Stationary Radiolysis

A mixture of BrSdU (10^−4^ M), 0.03 M *t*-BuOH (used as a scavenger of the ^•^OH radicals), and phosphate buffer (10 mM, pH = 7.0) was purging with argon for ca. 3 min in order to remove oxygen from the solution. The dose absorbed by all samples during irradiation was 140 Gy (4.14 Gy ∙ min^−1^, 130.0 kV, 5.0 mA). The studied samples were analyzed in triplicate. Radiolysis was performed in a Cellrad X-ray cabinet (Faxitron X-ray Corporation, Tucscon, AZ, USA).

#### 3.5.1. HPLC Analysis

Irradiated and non-irradiated samples of BrSdU were analyzed with reversed-phase HPLC method. For the separation of analytes a C18 column (Wakopak Handy ODS, 4.6 · 150 mm, 5 μm in particle size and 100 Å in pore size), gradient elution with 80% ACN and 0.1% HCOOH (from 0 to 50% ACN in 30 min), flow rate 1 mL · min^−1^ were used. The HPLC analysis was performed on a DionexUltiMate 3000 System (Dionex Corporation, Sunnyvale, CA, USA) with a Diode Array Detector set at 260 nm.

#### 3.5.2. LC-MS and LC-MS/MS Analysis

The solution of BrSdU containing *t*-BuOH and phosphate buffer was analyzed by LC-MS and LC-MS/MS methods, before and after irradiation. Conditions of separations: Kinetex column (Phenomenex, 1.7 μm, C18, 100 Å, 2.1 × 150 mm); flow rate 0.3 mL · min^−1^; a gradient elution with 80% ACN and 0.1% HCOOH (from 0 to 50% acetonitrile); the oven temperature was maintained at 25 °C. The effluent was diverted to waste for 2 min after injection. Conditions for MS and MS/MS analysis: the spray voltage was −4.5 kV, the nebulizer gas (N_2_) pressure was 25 psi, the flow rate was 11 L · min^−1^, and the source temperature was 300 °C. Each spectrum was obtained by averaging three scans and the time of each scan was 0.25 s. The LC-MS and LC-MS/MS analysis was performed TripleTOF 5600+ (SCIEX) mass spectrometer (operated in negative mode) coupled with Ultra-High Performance Liquid Chromatography (UHPLC) system Nexera X2 (Shimadzu, Canby, OR, USA).

### 3.6. Pulse Radiolysis

Pulse radiolysis experiments were performed with the INCT LAE 10 MeV linear electron accelerator with a typical pulse length of 8 ns. A detailed description of the experimental setup can be found in [45], along with the basic details of the equipment and the data collection system. Absorbed doses per pulse were on the order of 20 Gy (1 Gy = 1 J · kg^−1^). Dosimetry was based on the solutions saturated with nitrous oxide, containing 10^−2^ M KSCN, taking a radiation chemical yield of *G* = 0.635 μmol · J^−1^ and a molar absorption coefficient of 7580 M^−1^·cm^−1^ at 472 nm for the (SCN)_2_^•−^ radical [45]. Experiments were performed with a continuous flow of sample solutions at room temperature (~23 °C). All solutions were made with triply distilled water provided by a Millipore Direct-Q 3-UV system. The typical concentration of BrSdU and ISdU was 5 · 10^−5^ M, and 0.5 M of *t*-BuOH was used as a hydroxyl radical scavenger. Solutions were deoxygenated by purging with high purity argon, and 10 mM of phosphate buffer was added to maintain pH = 7. 

### 3.7. Clonogenic Assay

Adherent cell line MCF-7 (human breast cancer cells obtained from Cell Line Service–CLS, Eppelheim, Germany), which was treated with BrSdU in concentration of 10^−4^ and 10^−5^, respectively, was plated on 60 mm dishes in a density of 10^6^ cells per dish. After 48 h incubation under 37 °C and 5% CO_2_, the cells were exposed to 0.5, 1, 2, and 3 Gy, respectively (1.27 Gy ∙ min^−1^, 130.0 kV, 5.0 mA). After 6 h, the cells were trypsinized and plated on 100 mm dishes in a density of 800 cells per dish. After 16 days, formed colonies were fixed with 6.0% (*v*/*v*) glutaraldehyde and 0.5% crystal violet. Stained colonies were counted manually, and colony size was rated using inverted fluorescence microscope (Olympus, IX73, Tokyo, Japan). The cells were grown in the RPMI medium supplemented with 10% FBS (fetal bovine serum) and with antibiotics (streptomycin and penicillin) at concentration of 100 U · mL^−1^. Irradiation has been performed in a Cellrad X-ray cabinet (Faxitron X-ray Corporation). Plating efficiencies are shown in Table S1 in Supplementary Materials.

### 3.8. Cytotoxicity Assay

The MTT assay was used to identify the cytotoxic activity. Adherent MCF-7 and HDFa cell lines were seeded into 96-well plate in a density of 4 ∙ 10^3^ per well and incubated under 37 °C and 5% CO_2_, overnight. After that, the medium was replaced to fresh and the cells were treated with BrSdU at concentration of 0 (control), 10^−4^, 10^−5^, 10^−6^, 10^−7^, 10^−8^ M. Plates with cells were incubated (under the same conditions) with compound at 24 and 48 h. After this time, the aqueous solution of MTT salt at concentration 4 mg · mL^−1^ was added and incubate for 4 h. Then, the medium was removed and dimethylsulfoxide was added to each well in 200 µL volume. The absorbance was measured at 570 nm (and 660 nm, reference wavelength). Absorbance measurement has been performed with use of EnSpire microplate reader (PerkinElmer, Waltham, MA, USA). The liveliness of control was taken as 100%. The results were analyzed with the use of GraphPad Prism software. The statistical evaluation of treated samples and untreated control was calculated using one-way analysis of variance (ANOVA) followed by Dunnett’s multiple comparison test. The data was obtained from three independent experiments and each treatment condition assayed in triplicate. The differences were considered significant at *p* < 0.05. The cells were grown in the RPMI (MCF-7)/DMEM (HDFa) medium supplemented with 10% FBS (fetal bovine serum) and with antibiotics (streptomycin and penicillin) at concentration of 100 U · mL^−1^.

### 3.9. Incorporation of BrSdU and ISdU into Genomic DNA

The MCF-7 cell line was seeded into plate and incubated under 37 °C and 5% CO_2_ overnight. After that, the medium was replaced with fresh one and the cells were treated with BrSdU and ISdU at concentration of 0 (control) and 10^−4^ M. Plates with cells were incubated (under the same conditions) with compound for 48 h. After this time, the cells were pulled from the plates and isolation was carried out according to the protocol provided by the manufacturer (GeneMATRIX Cell Culture DNA Purification Kit, EURX, Gdańsk, Poland). After that, the purified DNA was enzymatically digested by the simultaneous action of DNase I, snake venom phosphodiesterase (SVP) and bacterial alkaline phosphatase (BAP).

#### 3.9.1. HPLC Analysis

The mixture of nucleoside (dC, dA, dG, dT, BrdU, BrSdU, and ISdU), nontreated and treated with BrSdU/ISdU samples, were analyzed with reversed-phase HPLC method. For the separation of analytes, a C18 column (Wakopak Handy ODS, 4.6 × 150 mm, 5 μm in particle size and 100 Å in pore size), gradient elution with 80% ACN, and 0.1% HCOOH (from 0 to 50% ACN in 30 min), flow rate 1 mL ∙ min^−1^ were used. The HPLC analysis was performed on the DionexUltiMate 3000 System with a Diode Array Detector (Dionex Corporation, Sunnyvale, CA, USA) set at 260 nm.

#### 3.9.2. LC-MS Analysis

DNA samples, isolated from the ISdU/BrSdU-treated culture, after enzymatic digestion were analyzed by LC-MS and LC-MS/MS methods. Conditions of separations: Kinetex column (Phenomenex, 1.7 μm, C18, 100 Å, 2.1 × 150 mm); flow rate 0.3 mL · min^−1^; a gradient elution with 80% ACN and 0.1% HCOOH (from 0 to 50% acetonitrile); the oven temperature was maintained at 25 °C. The effluent was diverted to waste for 2 min after injection. Conditions for MS and MS/MS analysis: the spray voltage was −4.5 kV and the source temperature was 300 °C. The LC-MS and LC-MS/MS analyses were performed with the use of TripleTOF 5600+ (SCIEX) mass spectrometer (operated in negative mode) coupled with Ultra High Performance Liquid Chromatography (UHPLC) system Nexera X2.

### 3.10. Flow Cytometry Analysis of Histone H2A.X Phosphorylation and CellDdeath

#### 3.10.1. Analysis of Histone H2A.X Phosphorylation

Human cells of breast cancer MCF-7 were grown in RPMI medium supplemented by the 10% FBS and antibiotics (streptomycin and penicillin) at a concentration of 100 U · mL^−1^. Cells, at a density of 0.2 ∙ 10^6^ per plate, were incubated for 24 h (37 °C, 5% CO_2_). After this time, the cells were treated with BrSdU at a concentration of 10^−4^ M and incubated for next 48 h under the same conditions. Then plate cultures were irradiated (Cellrad X-ray cabinet, Faxitron X-ray Corporation) with 1 and 2 Gy doses (1.27 Gy ∙ min^−1^, 130.0 kV, 5.0 mA) and incubated for 1 h. After this time, the MCF-7 cells were dissociated with 1x Accutase solution, fixed, permeabilized, and stained. The last step was cytometric analysis (Guava easyCyte™ 12, Merck, Hayward, CA, USA). Fixation, permeabilization, and staining were carried out according to the manufacturer’s protocol (FlowCellect™ Histone H2A.X Phosphorylation Assay Kit, Merck). The experiment was carried out in duplicate. Nontreated cultures were used as controls.

#### 3.10.2. Cell Death

After 24 h incubation, cells were treated with BrSdU at a concentration of 10^−4^ M and again incubated for 48 h (37 °C, 5% CO_2_). Then, the cells were irradiated (Cellrad X-ray cabinet, Faxitron X-ray Corporation) with a dose of 5 Gy (1.27 Gy ∙ min^−1^, 130.0 kV, 5.0 mA) and incubated for 24 h under the same conditions. After this time, the cells were dissociated with 1x Accutase solutions and analyzed by flow cytometry (Guava easyCyte™ 12, Merck, Warsaw, Poland) using the manufacturer’s protocol (FlowCellect™ MitoDamage Kit, Merck).

### 3.11. Computational

All calculations were performed with the B3LYP functional and DGDZVP++ basis set using the Gaussian09 package. Water environment was simulated with the polarizable continuum model (PCM) implemented therein, and in one of the reactions, three water molecules were included explicitly. In order to reduce the cost of calculations, 2′-deoxyribose moiety in nucleoside was substituted with the methyl group in the computational model (i.e., we used 5-bromo-1-methyl-4-thiouracil (BrSU), see Figure 2 and Figure 3) The sugar moiety does not take part in any of the considered reactions, hence the conversion of the studied system to BrSU is not expected to affect the computational results. The minima, as well as transition states (TSs), were proven with the vibrational frequency calculations, and the IRC procedure was applied to show that particular TSs are connected to the appropriate minima.

## 4. Conclusions

Radiotherapy is one of the most common modalities employed against cancer diseases. Unfortunately, its efficacy is seriously impaired due to hypoxia of solid tumors. Therefore, to be efficient, radiotherapy should be combined with radiosensitizers–compounds that are able to sensitize cells to ionizing radiation. The modified uridines belong to radiosensitizers, which produce radiosensitization by incorporation into DNA. Results of numerous studies, mainly on BrdU and IdU, suggest that dissociative electron attachment to the modified uridines incorporated into DNA is responsible for the increased level of damage. 

Our studies seem to confirm this view. Using enzymatic digestion of genomic DNA extracted from the cells incubated with BrSdU and ISdU, which was followed by the HPLC and LC-MS analysis of the lysates, we demonstrated that both compounds are incorporated into the DNA of the studied cell lines. Although BrSdU/ISdU are nontoxic, as shown by the results of the MTT test, their radiosensitizing efficacy turned out to be very different. Thus, clonogenic assay, the method of choice to determine cell reproductive death after treatment with ionizing radiation, shows significant activity of ISdU and practical lack of radiosensitization in the case of BrSdU. Similarly, the number of viable, early apoptotic, late apoptotic, and dead cells was not influenced by the pre-incubation with BrSdU, while the incubation with ISdU significantly increased the population of apoptotic and dead cells. Finally, the level of double-stand breaks which are associated with the cell death, assayed with the phosphorylation of histone γH2A.X test, clearly increased only in case of incubation with ISdU.

The significant variation in radiosensitizing activity of such similar derivatives has been explained with our radiolysis and computational studies. The stationary radiolysis of BrSdU demonstrated that although the compound was decomposed, no DEA products were detected. On the other hand, the radiolysis of ISdU solutions led to 4-thio-2’-deoxyuridine and its dimers. A similar conclusion was drawn from pulse radiolysis. Namely, the main dissimilarity between two studied 4-thio-2’-deoxyuridines lies in the fact that the transient absorption of 4SdU^●^ radical was observed only in the solutions of ISdU.

All of the above-mentioned observations are well-explained by difference in the activation barrier that accompanies the release of the halogen anion from the XSdU^●−^ anion radical. Namely, the barrier for BrSdU^●−^ is more than two-fold larger than that characteristic for DEA in ISdU^●−^. As a consequence, the C–X dissociation process is ca. 200-fold slower in the BrSdU anion. Hence, the lifetime of the anion is sufficiently long for BrdU^●−^ to be protonated forming BrSdUH^●^, which prevents the DEA process. Thus, our studies confirm the crucial role of DEA leading to uracil-5-yl radical in the radiosensitization mechanism of modified uracils. Moreover, they show that the relatively low barrier of ca. 26 kJ/mol is able to inhibit DEA in the studied class of molecules. Our studies demonstrate that even such a modest barrier makes the potential radiosensitizer completely inactive. This finding is of highly valuable for the computational search of radiosensitizing molecules.

## Supplementary Materials

The following are available online, LC-MS/MS identification of radiolysis products (Figures S1–S4); Transition state geometries for the reactions identified during stationary radiolysis (Figure S5); Incorporation yield of BrSdU and ISdU into the genomic DNA determined with its digestion and HPLC and LC-Ms analyses (Figures S6–S10); Flow cytometry analysis of histone H2A.X phosphorylation and cell death (Figures S11–S13).
