# Supplementary material for: Why Does the Type of Halogen Atom Matter for the Radiosensitizing Properties of 5-Halogen Substituted 4-Thio-2′-Deoxyuridines?
Source: Molecules. 2019 Aug 2;24(15):2819. doi: 10.3390/molecules24152819 (PMC6695862; doi:10.3390/molecules24152819)
Supplement: Supplementary file 1 [file molecules-24-02819-s001.pdf]

Supplementary Materials

Why Does the Type of Halogen Atom Matter for the Radiosensitizing Properties of 5-Halogen Substituted 4-Thio-2′-Deoxyuridines?

Paulina Spisz[1], Magdalena Zdrowowicz,[1] Samanta Makurat,[1] Witold Kozak,[1] Konrad Skotnicki,[2] Krzysztof Bobrowski,[2] Janusz Rak[1,*]

[1] Laboratory of Biological Sensitizers, Faculty of Chemistry, University of Gdańsk, Wita Stwosza 63, 80-308 Gdańsk, Poland; janusz.rak@ug.edu.pl

[2] Centre of Radiation Research and Technology, Institute of Nuclear Chemistry and Technology, Dorodna 16, 03-195 Warsaw, Poland; kris@ichtj.pl

* Correspondence: janusz.rak@ug.edu.pl

Table of content

Identification of radiolysis products (Figures S1-S4)	S3
Transition state geometries for reactions observed during stationary radiolysis (Figure S5)	S5
Incorporation of BrSdU and ISdU into the genomic DNA (Figures S6-S10)	S6
Clonogenic assay (Table S1)	S9
Flow cytometry analysis of histone H2A.X phosphorylation and cell death (Figures S11-S13)	S10

1. Identification of radiolysis products

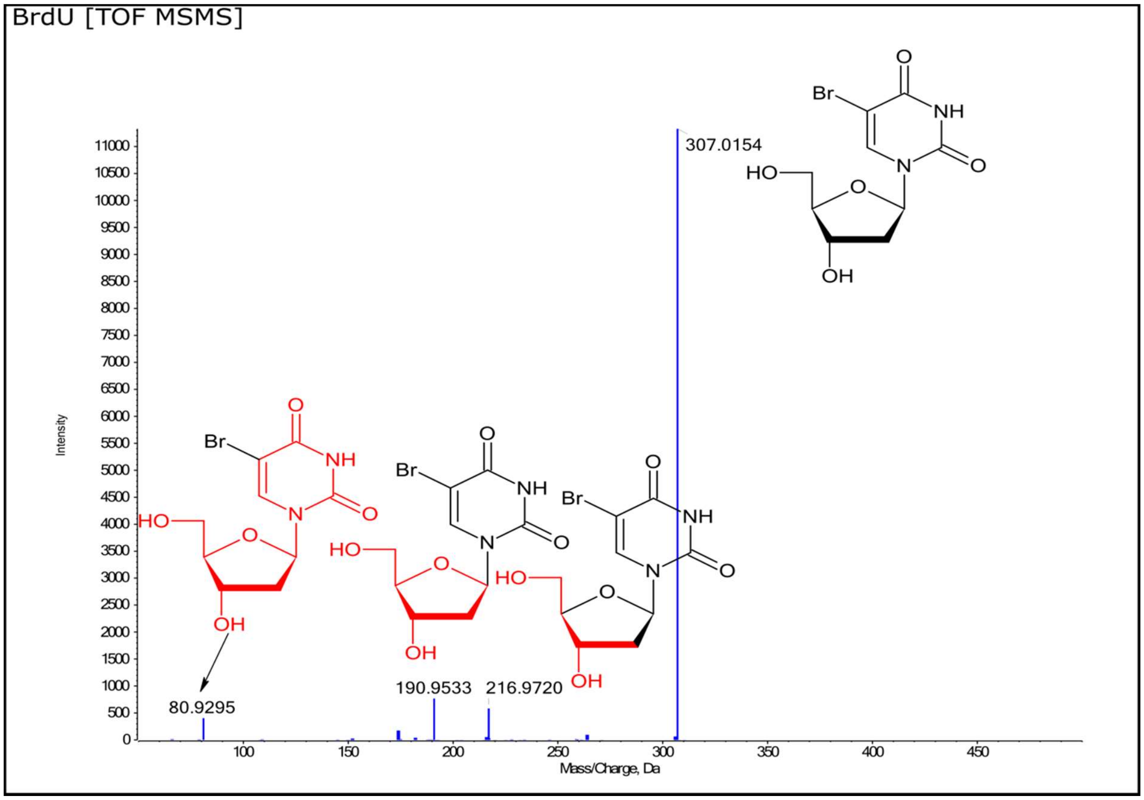

Figure S1. MS/MS spectrum and ion identities (in negative ionization mode) for BrdU.

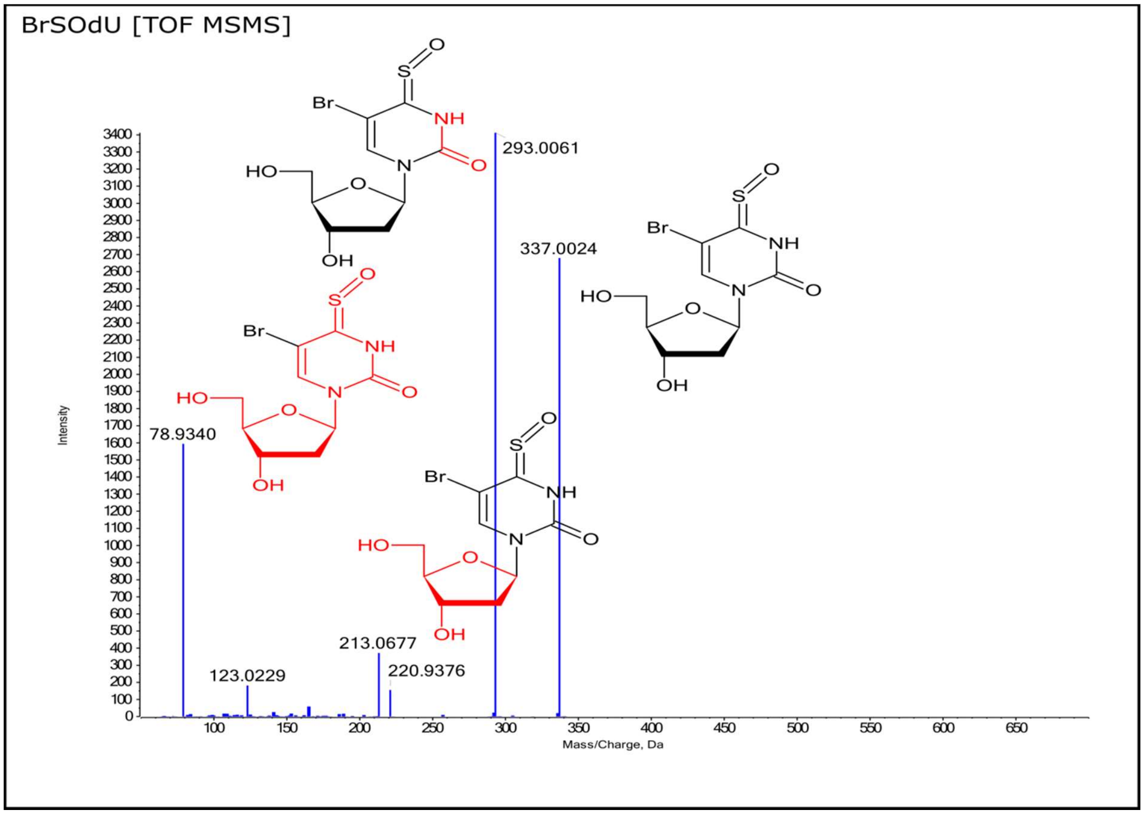

Figure S2. MS/MS spectrum and ion identities (in negative ionization mode) for BrSOdU.

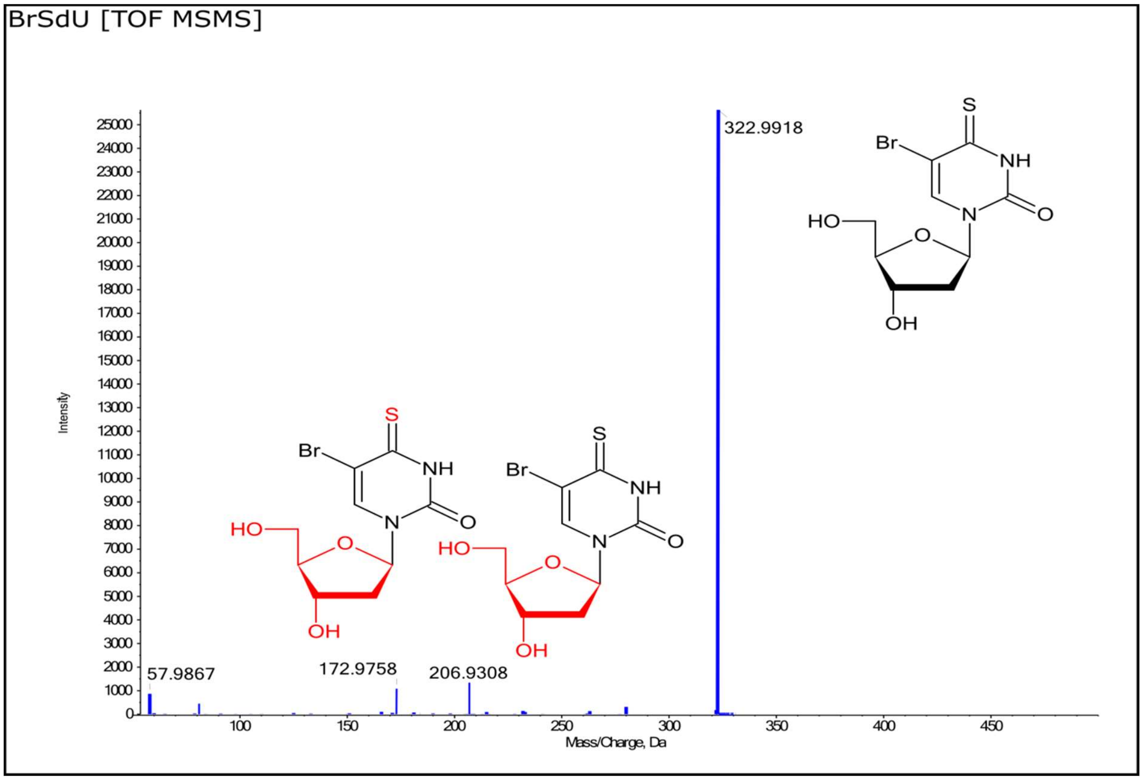

Figure S3. MS/MS spectrum and ion identities (in negative ionization mode) for BrSdU.

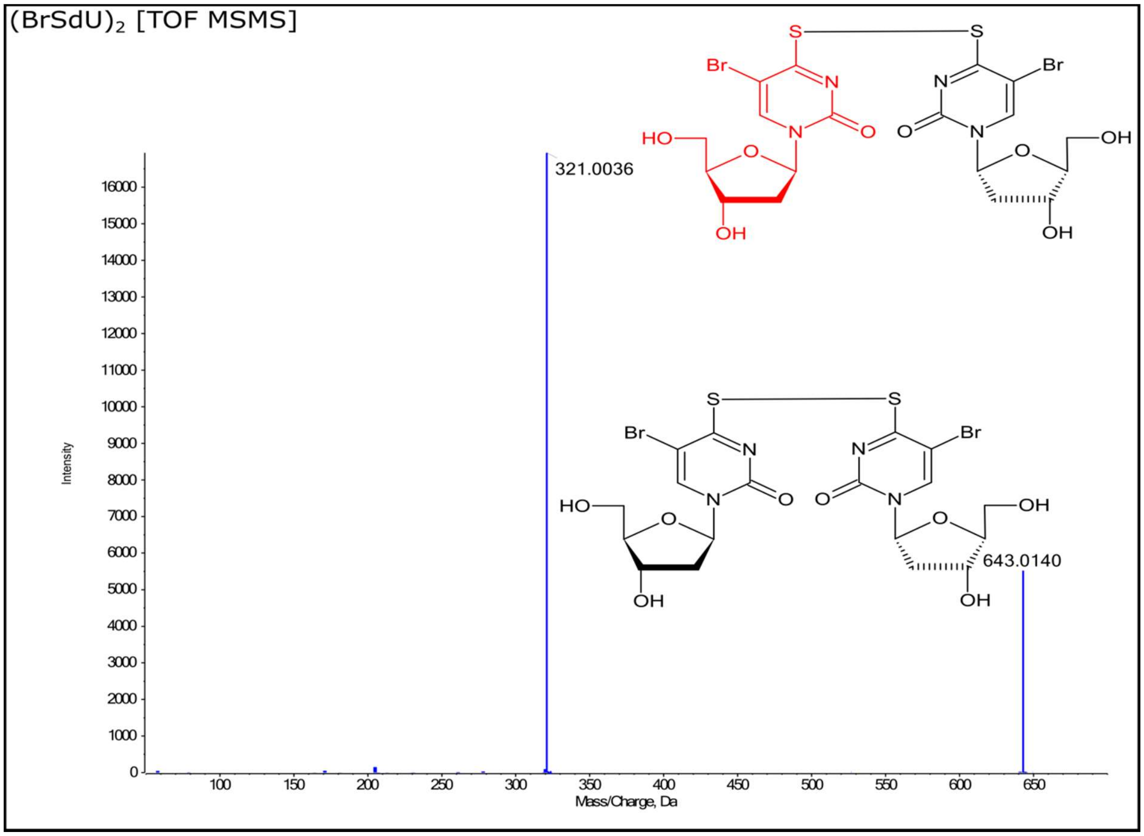

Figure S4. MS/MS spectrum and ion identities (in negative ionization mode) for $(BrSdU)_2$.

2. Transition state geometries for reactions observed during stationary radiolysis

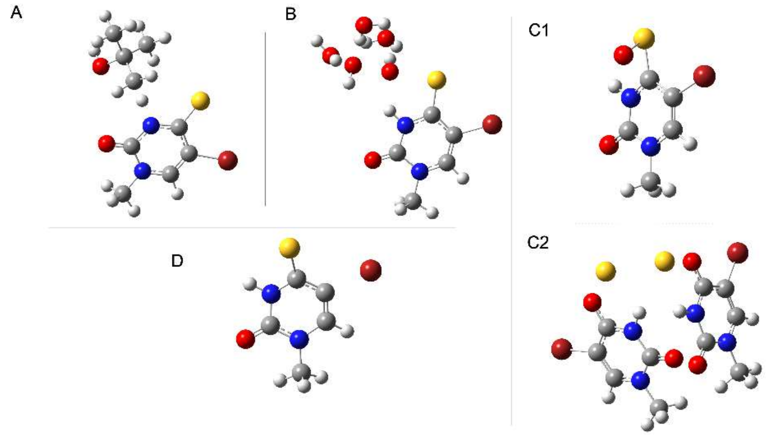

Figure S5. The transition state structures found in the computational studies: (A) $^{\bullet}CH_2(CH_3)_2COH$ with BrSU reaction (76.1 kJ/mol), (B) H_2O_2 with BrSU (74.1 kJ/mol), (C1) BrSOU to BrOSU transition (104.8 kJ/mol), (C2) 2BrOSU to 2BrU and S_2 reaction (4.4 kJ/mol). Additionally, the TS for DEA is shown (D, 26.0 kJ/mol).

3. Incorporation of BrSdU and ISdU into genomic DNA

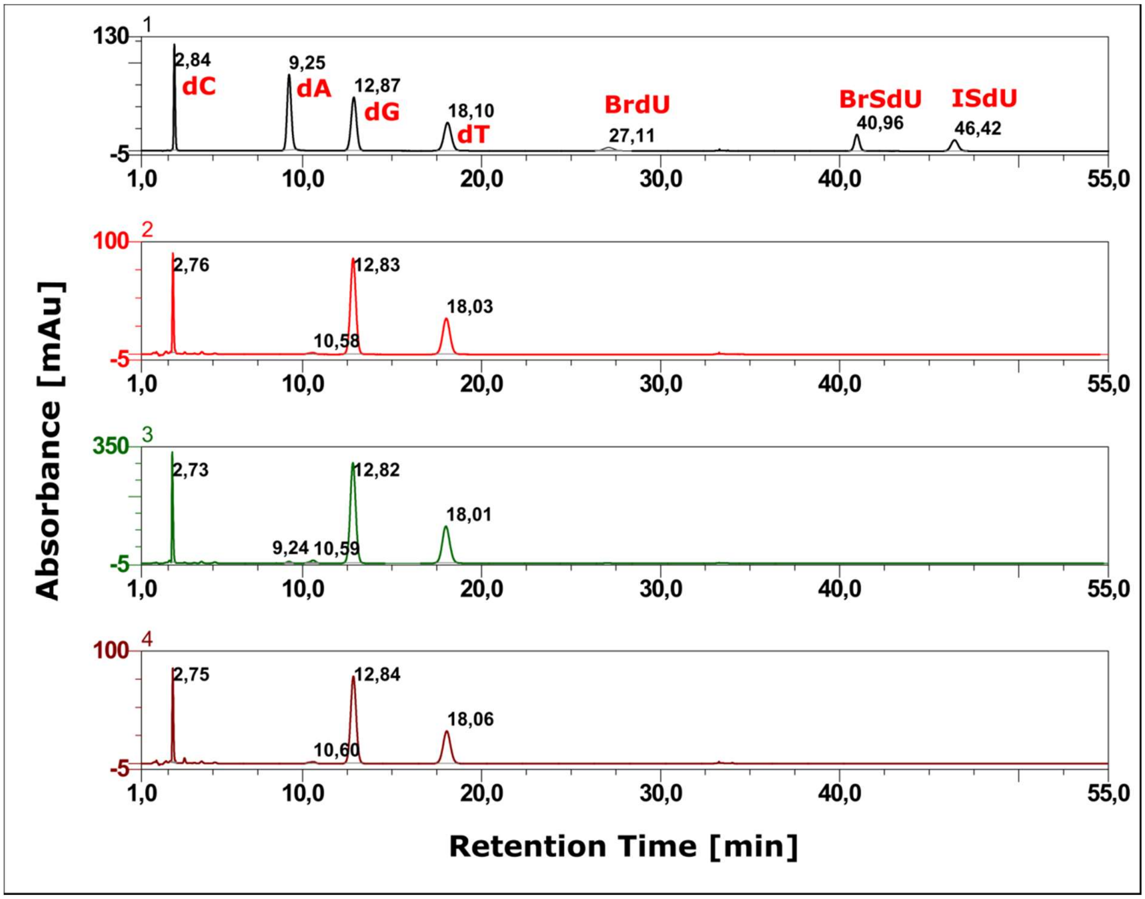

Figure S6. Incorporation analysis of BrSdU and ISdU. 1 – Mixture of nucleosides, 2 – Control (non-treated), 3 – Treated with BrSdU 10^{-4} M, 4 – Treated with ISdU 10^{-4} M.

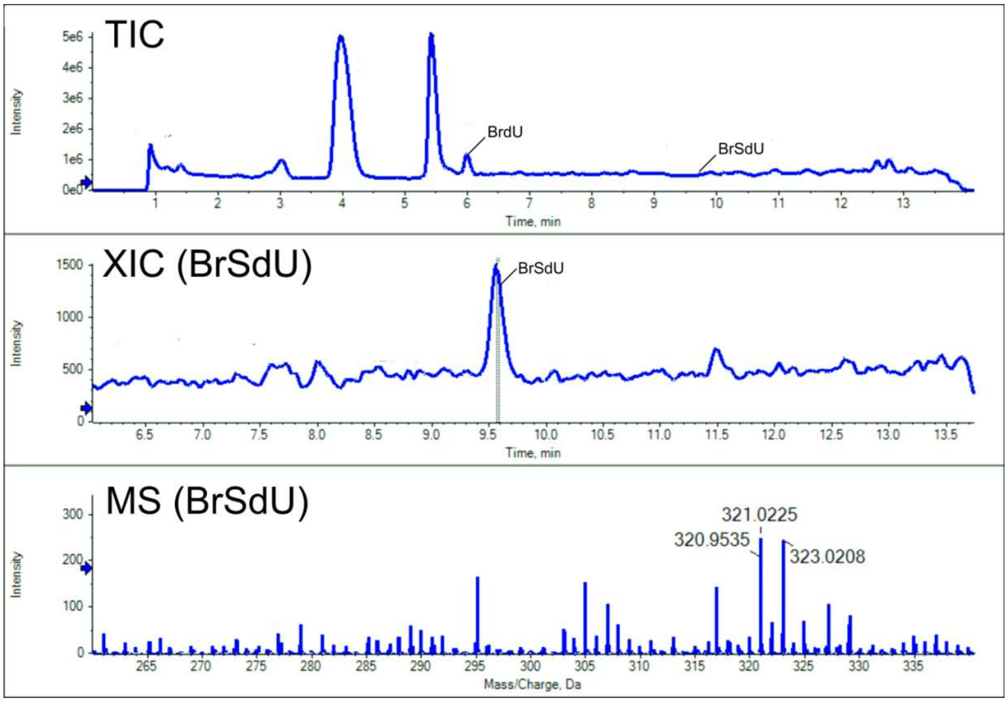

Figure S7. Incorporation of BrSdU into genomic DNA - LC-MS analysis. The upper panel: total ion current (TIC) for a DNA sample, isolated from a BrSdU-treated culture, after enzymatic digestion. The middle panel: extracted-ion chromatogram (XIC) for m/z 323 (ISdU). The lower panel: MS spectrum of BrSdU present in the digested sample.

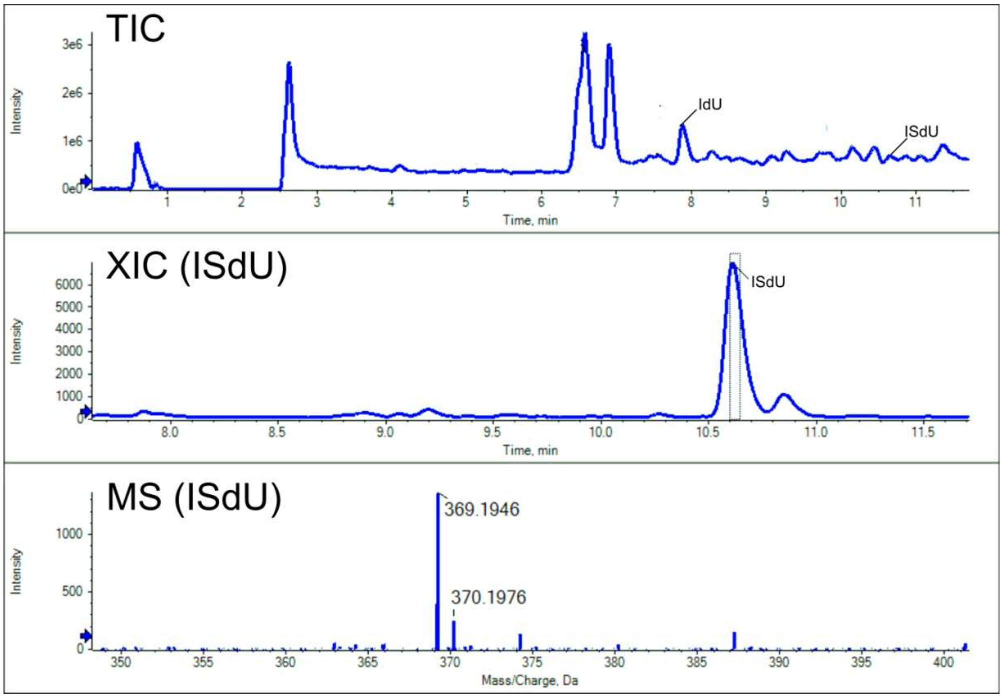

Figure S8. Incorporation of ISdU into genomic DNA - LC-MS analysis. The upper panel: total ion current (TIC) for a DNA sample, isolated from a ISdU-treated culture, after enzymatic digestion. The middle panel: extracted-ion chromatogram (XIC) for m/z 369 (ISdU). The lower panel: MS spectrum of ISdU present in the digested sample.

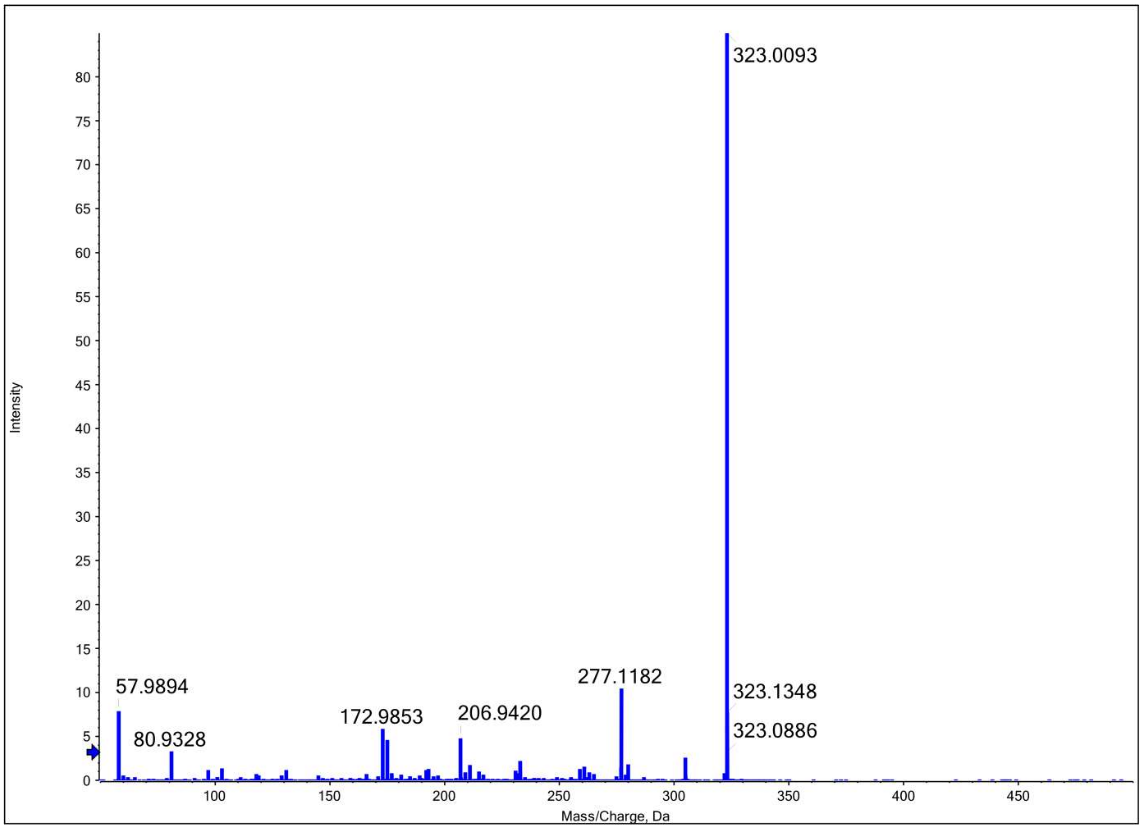

Figure S9. MS/MS spectrum of BrSdU present in the digested DNA sample, isolated from a BrSdU-treated culture.

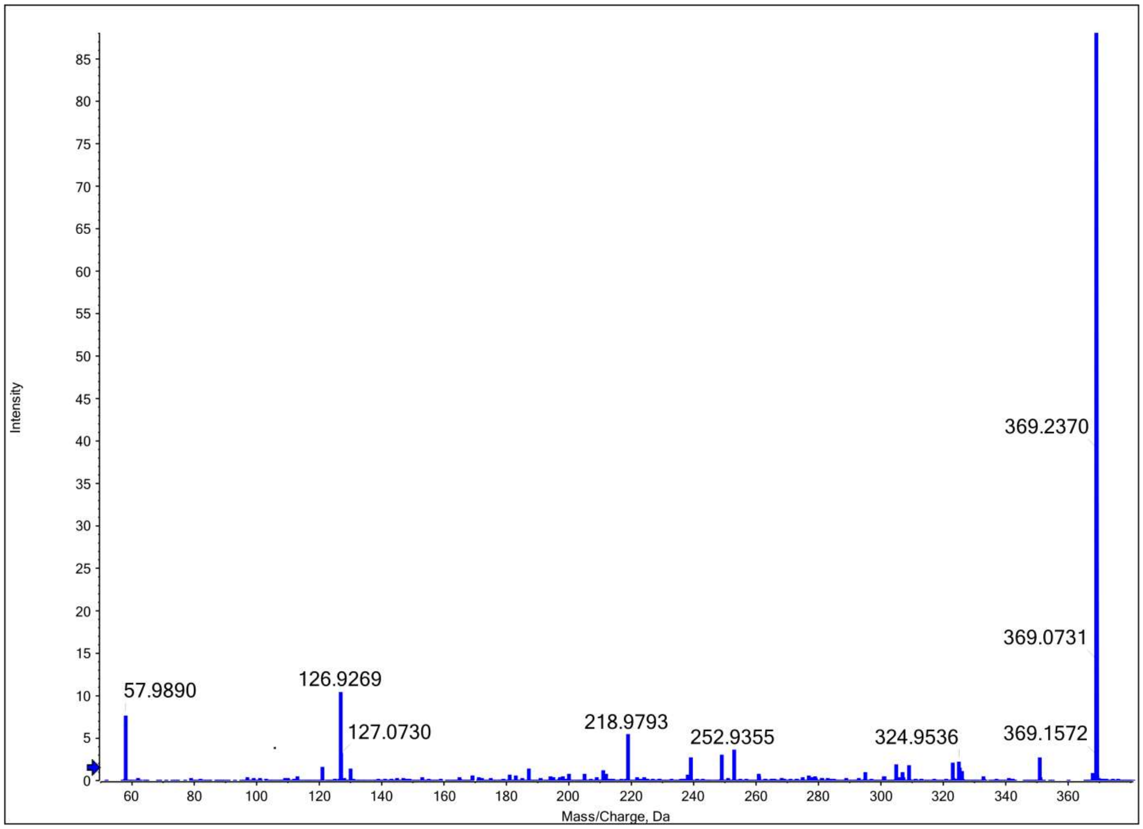

Figure S10. MS/MS spectrum of ISdU present in the digested DNA sample, isolated from a ISdU-treated culture.

4. Clonogenic assay

Table S1. Plating efficiencies [%] for the MCF-7 cells obtained from clonogenic assay.

	PLATING EFFICIENCIES [%]		
**Dose [Gy]**	0 μM BrSdU	10 μM BrSdU	100 μM BrSdU
**0**	28.16	25.63	24.78
**0.5**	17.66	17.31	16.63
**1**	14.00	13.63	12.06
**2**	8.69	8.75	7.09
**3**	4.69	3.56	2.94

5. Analysis of histone H2A.X phosphorylation and cell death

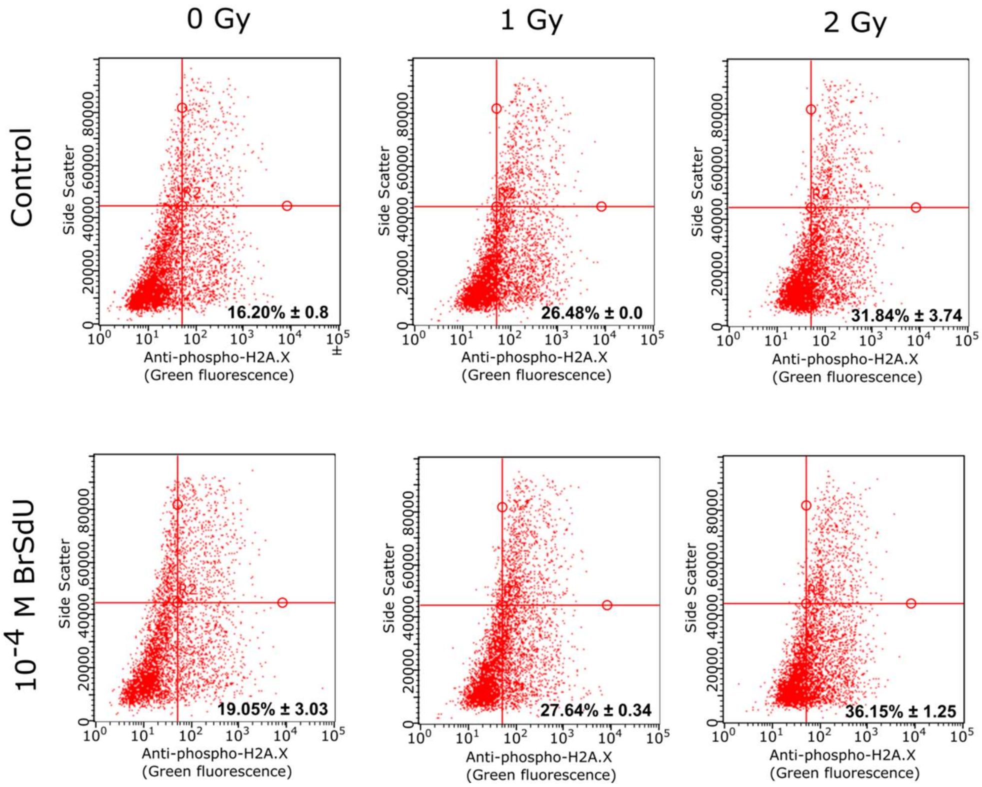

Figure S11. Flow cytometry analysis of histone H2A.X phosphorylation.

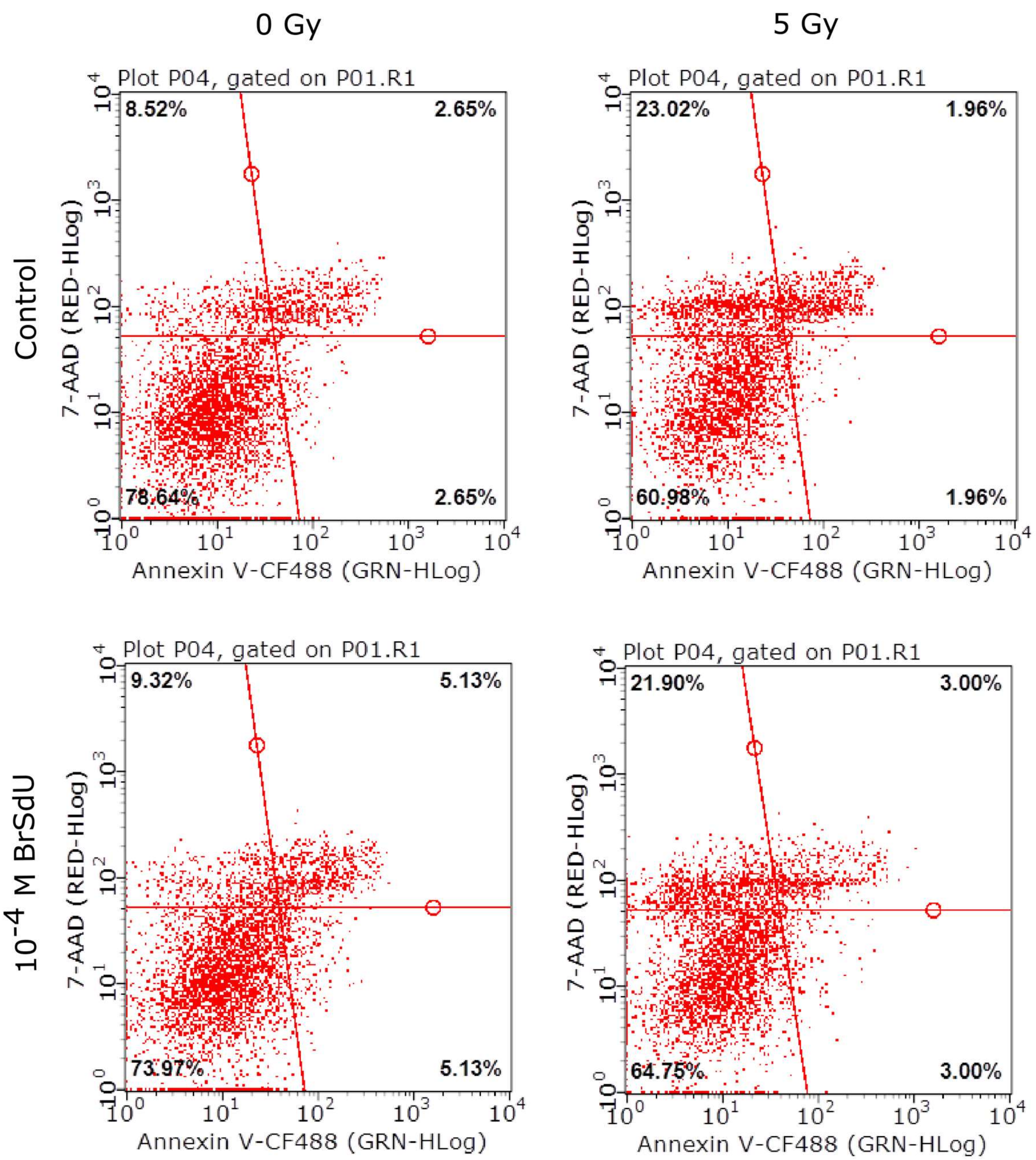

Figure S12. Flow cytometry analysis of cell death – dot plots provide comparison of 7-AAD (cell death) vs. Annexin V (late apoptosis).

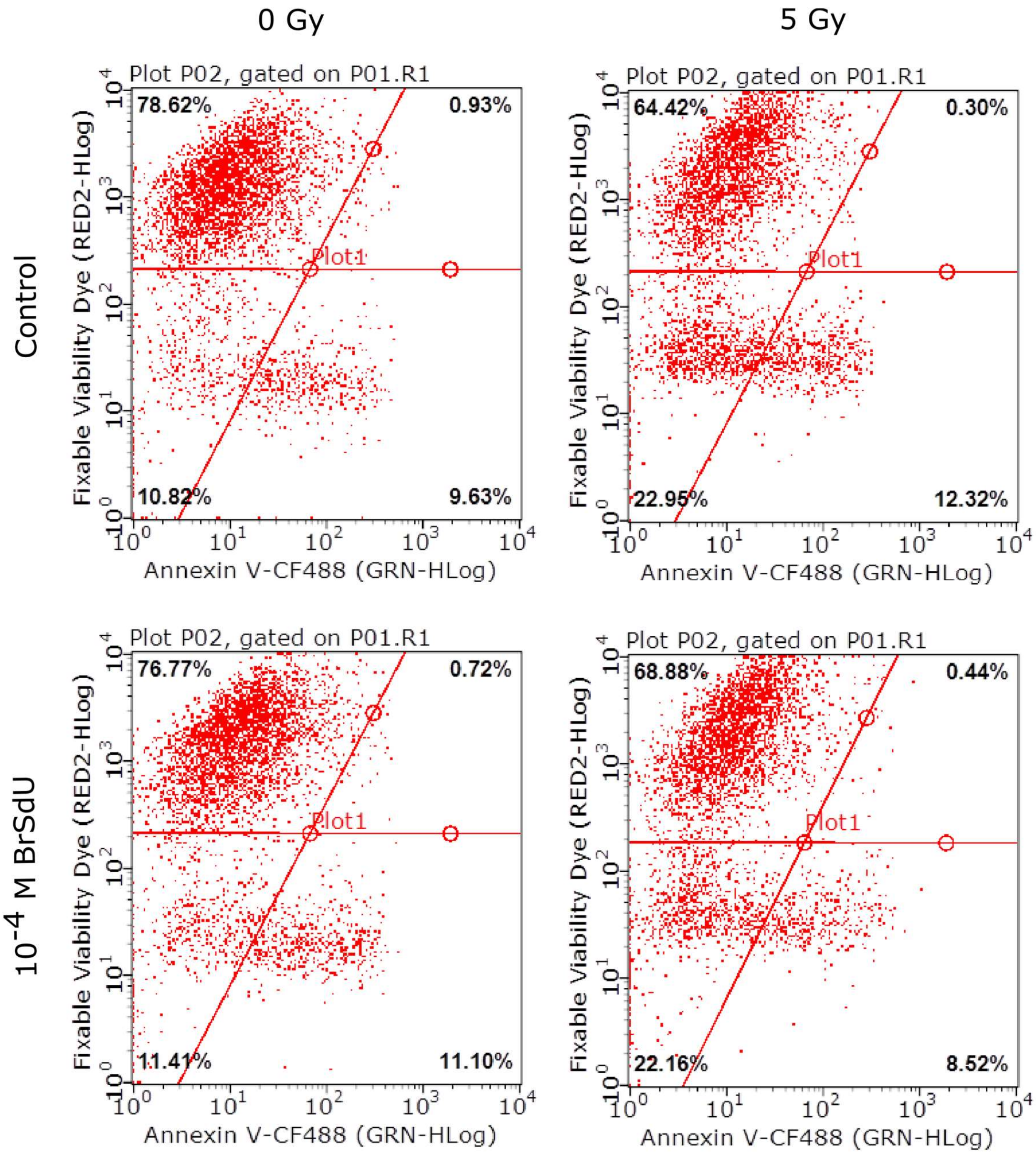

Figure S13. Flow cytometry analysis of cell death – dot plots provide comparison of MitoSense Dye (early apoptosis) vs. Annexin V (late apoptosis).
